# The immunorecognition, subcellular compartmentalization, and physicochemical properties of nucleic acid nanoparticles can be controlled by composition modification

**DOI:** 10.1093/nar/gkaa908

**Published:** 2020-10-22

**Authors:** Morgan Brittany Johnson, Justin R Halman, Daniel K Miller, Joseph S Cooper, Emil F Khisamutdinov, Ian Marriott, Kirill A Afonin

**Affiliations:** Department of Biological Sciences, University of North Carolina at Charlotte, Charlotte, NC, USA; Nanoscale Science Program, Department of Chemistry, University of North Carolina at Charlotte, Charlotte, NC 28223, USA; Department of Chemistry, Ball State University, Muncie, IN 47306, USA; Department of Chemistry, Ball State University, Muncie, IN 47306, USA; Department of Chemistry, Ball State University, Muncie, IN 47306, USA; Department of Biological Sciences, University of North Carolina at Charlotte, Charlotte, NC, USA; Nanoscale Science Program, Department of Chemistry, University of North Carolina at Charlotte, Charlotte, NC 28223, USA

## Abstract

Nucleic acid nanoparticles (NANPs) have become powerful new platforms as therapeutic and diagnostic tools due to the innate biological ability of nucleic acids to identify target molecules or silence genes involved in disease pathways. However, the clinical application of NANPs has been limited by factors such as chemical instability, inefficient intracellular delivery, and the triggering of detrimental inflammatory responses following innate immune recognition of nucleic acids. Here, we have studied the effects of altering the chemical composition of a circumscribed panel of NANPs that share the same connectivity, shape, size, charge and sequences. We show that replacing RNA strands with either DNA or chemical analogs increases the enzymatic and thermodynamic stability of NANPs. Furthermore, we have found that such composition changes affect delivery efficiency and determine subcellular localization, effects that could permit the targeted delivery of NANP-based therapeutics and diagnostics. Importantly, we have determined that altering NANP composition can dictate the degree and mechanisms by which cell immune responses are initiated. While RNA NANPs trigger both TLR7 and RIG-I mediated cytokine and interferon production, DNA NANPs stimulate minimal immune activation. Importantly, incorporation of 2′F modifications abrogates RNA NANP activation of TLR7 but permits RIG-I dependent immune responses. Furthermore, 2′F modifications of DNA NANPs significantly enhances RIG-I mediated production of both proinflammatory cytokines and interferons. Collectively this indicates that off-target effects may be reduced and/or desirable immune responses evoked based upon NANPs modifications. Together, our studies show that NANP composition provides a simple way of controlling the immunostimulatory potential, and physicochemical and delivery characteristics, of such platforms.

## INTRODUCTION

Nucleic acids play a plethora of critical biological roles in all forms of life that include serving as the genetic material, performing key functions in protein synthesis and the regulation of gene expression and editing. Rationally designed therapeutic nucleic acids (TNAs) exploit these properties to identify, target, and silence genes or cellular pathways to treat a wide range of disorders (1,2). In addition, TNAs are biocompatible and have highly programmable and tunable physicochemical characteristics (3–7). Despite these obvious benefits, the clinical use of TNAs remains limited due to several challenges. First, while RNA serves as an ideal building block for biologics because it can function as a ribozyme, a riboswitch, and as a regulator of gene expression, RNA based-nanoparticles have low serum stability (3,7,8). Second, NANP bioavailability remains a challenge as the negatively charged phosphate backbone of nucleic acids can prevent entry across similarly charged cell membranes (7). Therefore, NANPs must either be complexed with a carrier or functionalized with RNA aptamers to enter target cells (4,9–11). Furthermore, once inside, the functionalized NANPs must traffic to appropriate and specific subcellular locations. Third, NANPs can trigger severe off-target inflammatory responses (8,12) due to the presence of pattern recognition receptors that identify pathogen and damage associated molecular patterns (PAMPs and DAMPS, respectively), or can inappropriately modify innate and adaptive immune functions by serving as agonists for nucleic acid immunosensors (6,13–20). It is for this reason that NANPs that specifically activate nucleic acid immune sensors are considered promising candidates as pan-antivirals or vaccine adjuvants. As such, major research efforts have focused on the rational optimization of NANP structures and compositions to overcome these hurdles and balance the desired therapeutic outcome with their half-life, delivery, and immunostimulatory potential.

We have previously demonstrated the self-assembly of NANPs composed of RNA, DNA or a hybrid of both (3,21). Predictive quantitative structure–activity relationship modeling shows that the physicochemical properties of NANPs, including molecular weight, melting temperature, and half-life that are determined by the ratio of RNA to DNA, strongly correlate with their immunostimulatory properties (21). As such, the nucleic acid composition can be optimized to achieve the desired physicochemical and immunostimulatory properties. Additionally, chemically modified nucleic acid analogs can also be incorporated to optimize thermostability, serum stability, and immunostimulatory activity (3,5,7,14,22–26).

**Figure 1.**
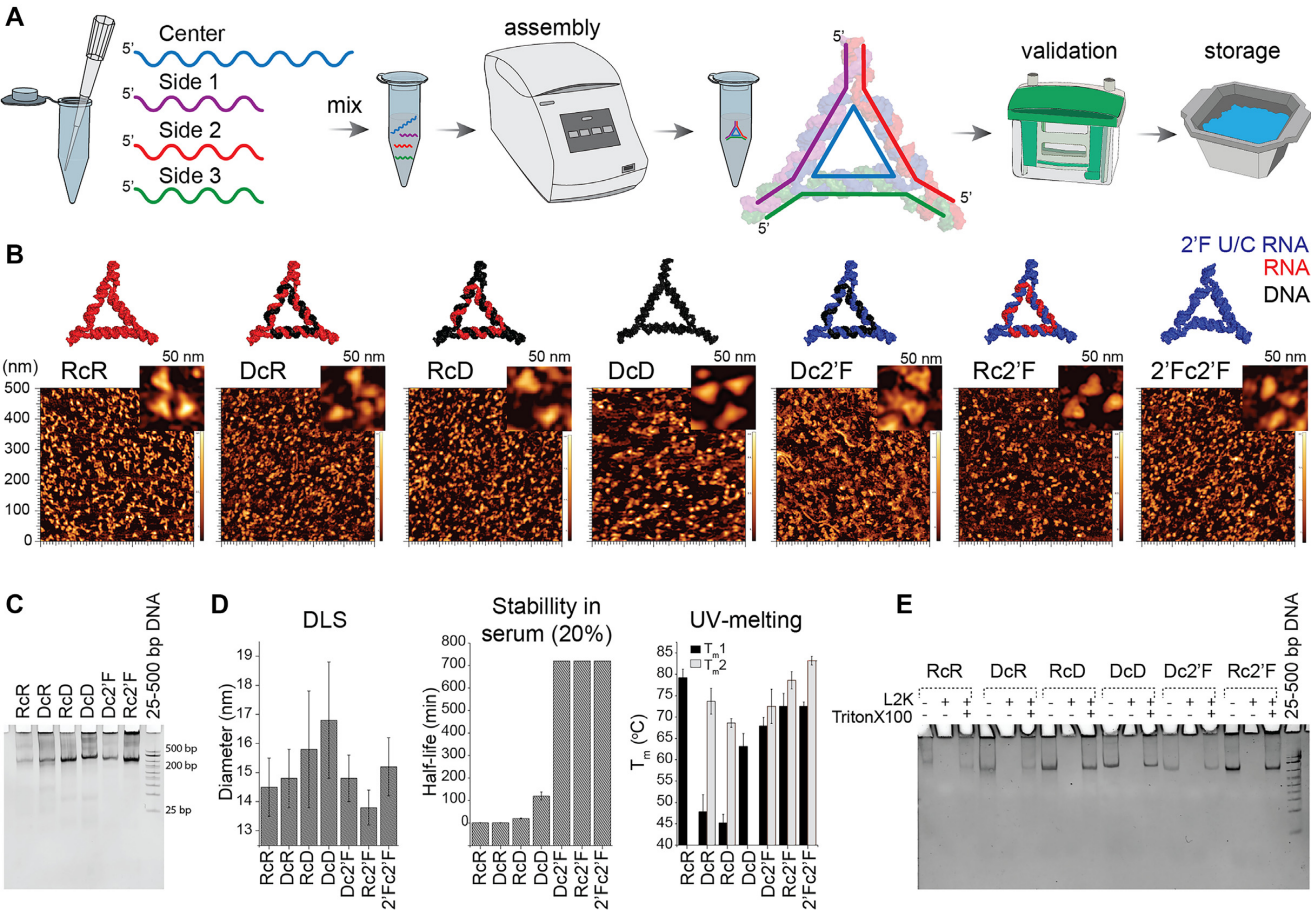
Assembly of NANPs and their physicochemical characterization. (**A**) A schematic of the assembly, conformation, and validation of NANPs. (**B**) Energy minimized 3D models of the NANPs and their corresponding AFM images. (**C**) Confirmation of assembly confirmed by EtBr total staining native-PAGE (representative gel). (**D**) Relative sizes of NANPs assessed by DLS, chemical stability of NANPs incubated in 20% FBS solution at 37°C from 1 to 720 min analyzed by EMSA, and melting temperatures assessed by UV-melting experiment. (**E**) Structural integrity of NANPs upon release from Lipofectamine 2000 (L2K) complexation confirmed by EtBr total staining native-PAGE (representative gel). Data are represented as the mean ± (SEM) for a minimum of three independent experimental replicates. The error for *T*_m_ and blood stability did not exceeded 10%. For C and E, 25–500 bp Low Range DNA Marker A (Biobasic) was used as the control.

In the present study, we have built upon our previous work to characterize a circumscribed panel of NANPs (Figure 1) that are composed of RNA, DNA and 2′F-modified oligonucleotides. We demonstrate that NANPs constructed with the same sequences, connectivity, shape, size, and charge, but that differ in chemical composition, exhibit marked differences in their physicochemical properties, subcellular accumulation, and immunostimulatory potential. Specifically, incorporation of 2′F modified strands increases the melting temperature and significantly enhances the serum stability of RNA and DNA NANPs. In addition, we demonstrate the successful intracellular delivery of NANPs *via* an endocytic pathway using lipid-based carriers and show that specific NANP-carrier combinations affect delivery efficiency and subcellular targeting. Finally, our data indicates that NANP chemical composition modification can be used to trigger or abrogate recognition by innate pattern recognition receptors to either mitigate detrimental inflammation or promote desirable immune responses.

## MATERIALS AND METHODS

### NANP synthesis

The synthetic oligonucleotides with 2′-fluoro modified pyrimidines (2′F-U/C) were purchased from Exonano RNA LLC. All DNA strands including 5′-end cy-3 labeled ODNs were purchased from Integrated DNA Technologies, Inc. DNA templates for *in vivo* transcription of 2′F-U/C modified RNA strands were amplified by PCR. The 2′F-modified RNA strand 2′F-T1 was prepared by 2′F *in vitro* T7 RNA transcription kit (Exonano RNA LLC) and purified by 8 M urea 18% PAGE. The corresponding bands were excised under UV shadow and eluted from the gel for 4 h at 37°C in elution buffer (Tris–HCl buffer (89 mM pH 8.0) 0.3 M NaOAc, 0.1 mM EDTA), followed by ethanol precipitation overnight at –20°C (2.5× volume of 100% ethanol and 1/10 volume of 3M NaOAc). The precipitate was pelleted by centrifugation (16 500 × g, 30 min), washed with 80% ethanol, and dried by speed vacuum. Finally, the 2′F-RNA dried pellet was rehydrated in double deionized water and stored at –20°C. All RNA, DNA, and fluorinated structures, were assembled by mixing corresponding nucleic acid strands (1 μM final) in assembly buffer (tris-borate buffer (89 mM, pH 8.3), 2 mM Mg(OAc)_2_ and 50 mM KCl) by heating the mixture to 100°C and slow cooling to 4°C. The annealing process for 2′F-RNA/DNA and 2′F-only NANPs occurred by slow cooling (1°C/ min) from 90°C to 4°C in a PCR thermal controller. The one-pot self-assembly protocol using equimolar concentrations resulted in high assembly efficiency across all NANPs. The purity of each batch of NANPs was evaluated by AFM imaging and native-PAGE gel and only batches with 90%+ yields were used in these studies.

### UV-melting experiments

The assembled NANPs (*C* = 0.1–0.2 μM, *V* = 100 μl in 1× AB) were degassed for 5 min using a speed-vac. The resulting mixture volume (∼100 μl) were placed into UV-melting cells (micro-cuvette Starna Cells, 10 mm path length) equipped with a PTFE stopper. Melting profiles were obtained using an Agilent spectrophotometer at 260 nm over a temperature range of 20–100°C at a ramp rate of 0.1°C/min. All UV melting assays were performed three times. The error for *T*_m_ did not exceed 10%. The nonlinear dose response function of Origin Pro was used to fit absorbance data according to the following equation:

$$\begin{equation*}{{y}} = {{A}}\_1 + \frac{{{{A}}\_2-{{A}}\_1}}{{1 + {{10}^{\left( {{\rm{LOGX}\_0 - X}} \right){{P}}}}}}\end{equation*}$$

where *X* is the temperature (°C); *y* is absorbance at λ = 260 nm; *A*_1 is the bottom plateau (asymptote 1) or Abs at fully associate state; *A*_2 is the is the upper plateau (asymptote 2) or Abs at fully dissociated state; LOGx_0 is the temperature at the midpoint or *T*_m_; *p* is a measure of steepness of the inflection.

### Dynamic light scattering analysis

The assembled NANPs (*C* = 1 μM, *V* = 100 μl in 1× AB) were filtered through 50 kDa Ultracel-50 regenerated cellulose membrane (MWCO) at 12 000 × g for 2.5 min. The remaining volumes (∼100 μl) were transferred to DLS cells (micro-cuvette, Starna Cells) and analyzed at 25°C using a Zetasizer nano-ZS (Malvern Instrument Ltd).

### Fetal bovine serum stability assay

The assembled NANPs (1 μM in AB pH 8.0) were incubated in 20% fetal bovine serum (FBS) solution. Aliquots were taken at time intervals ranging from 1 min to 12 h and the remaining particle fractions were evaluated using 3% agarose gel electrophoresis in two experiments. As a control, corresponding NANPs were incubated in buffer for 12 h at 37°C in the absence of FBS. ImageJ software was used to quantify the remaining fraction of NANPs from the gel image. The resulting fraction percentage was plotted as a function of incubation time using Origin Pro software.

### Integrity of NANP upon release from a carrier

The assembled NANPs (100 nM) were complexed to Lipofectamine 2000 (L2K) by mixing and incubating at room temperature at a final volume of 9 μl. After 30 min, 1 μl of 10% Triton-X was added, and the solution was incubated for another 30 min. Samples were then separated by native-PAGE, (37.5:1, 8%) and visualized with ethidium bromide staining.

### Source and propagation of cell lines

We selected immortalized primary human microglia and the THP monocyte-like cell line for use in these studies as they are models for tissue specific and circulatory myeloid immune cells, respectively, and such cells are recognized to play a pivotal role in acute local and systemic inflammatory responses to microbial and damage associated molecular motifs, including nucleic acids. The human microglia cell line, hμglia, was a generous gift from Dr Jonathan Karn (Case Western Reserve University). Primary human cells were transformed with lentiviral vectors expressing SV40 T antigen and hTERT. These cells were classified as microglia based on microglia-like morphology, migratory and phagocytic activity, presence of microglia cell surface markers CD11b, TGFβR and P2RY12, and a characteristic microglial RNA expression profile (3,21,27–30). This cell line was maintained in Dulbecco's modified Eagle's medium supplemented with 5% FBS and penicillin/streptomycin (100 U/ml–100 μg/ml). THP-1-Dual Cells were purchased from InvivoGen. These cells were derived from the THP-1 monocyte cell line by stable integration of two inducible reporter constructs to monitor the NF-κB and IRF pathways. Cells were maintained according to the supplier's guidelines in RPMI 1640, 2 mM glutamine, 25 mM HEPES, 10% heat-inactivated FBS, 100 μg/ml normocin, penicillin/streptomycin (100 U/ml and 100 μg/ml). Reporter proteins were measured using a secreted embryonic alkaline phosphatase (SEAP) detection reagent, QUANTI-Blue, and a luciferase detection reagent, QUANT-Luc. HEK TLR Reporter Cells were purchased from Invivogen. These cells were generated from a human embryonic kidney cell line, HEK293, co-transfected with either the human TLR3, TLR7 or TLR9, genes and an inducible SEAP reporter gene. The cell culture medium HEK-Blue detection was used to determine levels of SEAP activity.

### Reporter cell lines

THP1-Dual and HEK-Blue reporter cell lines (Invivogen) were seeded at 4 × 10^5^ cells per well in a 96-well-plate and either allowed to adhere overnight or used immediately. NANPs were incubated for 30 min with L2K prior to transfection of reporter cell lines with NANPs (5 nM). Cells were incubated with the transfection reaction for 24 h. For the THP1-Dual, HEK-Blue hTLR 3, 7 and 9, cells, 20 μl of cell supernatant was mixed with 180 μl of Quanti-Blue in a 96-well-plate and incubated at 37°C for up to 3 h and the absorbance read at 620 nm. For THP1-Dual and HEK-Lucia RIG-I cells, 20 μl of cell supernatant was mixed with 50 μl of Quanti-Luc in a black-walled 96-well plate, and the luminescence was read immediately.

### Transfection of Microglia

The hμglia cell line was transfected using Lipofectamine 2000 or L2K (Invitrogen) or DOTAP (Milipore Sigma) according to the manufacturer's guidelines. NANPs were incubated for 30 min with L2K or DOTAP prior to transfection of hμglia with nucleic acid nanoparticles (5 nM) for 4 h in DMEM supplemented with 5% FBS. The cell culture media was subsequently changed to media supplemented with 100 U/ml penicillin-100 μg/ml streptomycin and cell supernatants were collected for analysis at the indicated time points.

### siRNA knockdown

The hμglia cell line was transfected with 5 nM control siRNA (silencer select negative control number 1 siRNA, ThermoFisher Scientific), siRNA targeting RIG-I (αRIG-I) (ThermoFisher Scientific assay identification number s223615), or siRNA targeting RNA polymerase III subunit A (ThermoFisher Scientific assay identification number s21945), according to the manufacturer's guidelines using RNAimax (ThermoFisher Scientific). Cells were transfected for 48 h prior to transfection with nanoparticles as described above. Cell lysates and supernatants were collected for analysis at the indicated time points.

### Quantification of cytokines in cell supernatants

Specific capture ELISA were performed to quantify human IL-6 and IFN-β production. A rat anti-human IL-6 capture antibody (BD Pharmingen, cat# 554543, Clone Mq2-13A5) and a biotinylated rat anti-human IL-6 detection antibody (BD Pharmingen, cat# 554546, Clone MQ2-39C3) were used for IL-6 capture ELISAs. A polyclonal rabbit anti-human IFN-β capture antibody (Abcam, cat# ab186669) and a biotinylated polyclonal rabbit anti-human IFN-β detection antibody (Abcam, cat# ab84258) were used in IFN-β capture ELISAs. Streptavidin–horseradish peroxidase (HRP) (BD Biosciences) was added prior to the addition of tetramethylbenzidine substrate to detect bound antibody. The reaction was stopped using H_2_SO_4_ and the absorbance was measured at 450 nm. Recombinant cytokines for IL-6 (BD Pharmingen) or IFN-β (Abcam) were diluted to generate standard curves and the cytokine concentration in cell supernatants was determined by extrapolation of absorbance to the standard curve.

### Immunoblot analyses

Cell lysates were evaluated for the expression of RIG-I and RNA polymerase III subunit A by immunoblot analyses. Blots were incubated with a rabbit polyclonal antibody against RIG-I (Abgent, cat# AP1900a), a rabbit monoclonal antibody against RNA polymerase III subunit A (Cell signaling, cat# 12825S, clone D5Y2D), a rabbit monoclonal antibody against GAPDH (Cell Signaling, cat# 5174S), a rabbit monoclonal antibody against histone 3 (H3; Cell Signaling, cat# 4499S), or a rabbit monoclonal antibody against COX IV (Cell Signaling, cat# 9367), overnight at 4°C. Blots were washed and incubated in the presence of a HRP-conjugated secondary anti-rabbit antibody. Bound antibody was detected with the WesternBright ECL kit (Advansta). Immunoblots were then reprobed with a mouse monoclonal antibody against β-actin (Abcam, cat# 49900) to assess total protein loading. Immunoblots are representative of at least three separate experiments.

### Cellular fractionation

Following transfection with Cy3 labeled nanoparticles for 2 or 4 h, a cellular fractionation kit (Cell Signaling) was used to separate cells into three fractions: cytosolic (cytosolic isolation buffer; CIB), membrane/organelle (membrane/organelle isolation buffer; MIB), and nuclear/cytoskeleton (nuclear/cytoskeleton isolation buffer; NIB). The separation of cell fractions was carried out according to the manufacturer guidelines. The purity of these fractions was determined via immunoblot for proteins GAPDH, COX IV, Rab7 and H3, as described in the immunoblot section. The fluorescence (Ex540/Em580) of each fraction was evaluated using a SpectraMax iD5 plate reader.

### Flow cytometric analysis

The microglia cell line, hμglia, was transfected with 5 nM Cy3-labeled NANPs using either L2K or DOTAP. After 4 h, cells were removed from tissue culture plates using 0.05% trypsin and fixed with 1% paraformaldehyde prior to flow cytometric analysis using an Accuri C6 cytometer (BD Biosciences, Franklin Lakes, NJ, USA) to evaluate NANP uptake.

### Fluorescent immunohistochemical analysis

The microglia cell line, hμglia, were plated on poly-d-lysine coated glass coverslips and cells were transfected with 5 nM Cy3-labled NANPs using either L2K or DOTAP. After 4 h, cells were fixed with 4% paraformaldehyde, permeabilized with 0.1% Triton-X-100, and blocked (2% BSA). Cells were stained with a monoclonal rabbit antibody directed against EEA1 (Invitrogen, clone F.43.1) followed by incubation with a polyclonal goat anti-rabbit secondary antibody coupled to Alexa Fluor 647 (Invitrogen). Samples were mounted with Prolong Diamond antifade mountant with DAPI (Invitrogen) and imaged using an Olympus Fluoview 1000 four-color confocal laser microscope.

### Statistical analysis

Data is presented as the mean ± standard error of the mean (SEM). Statistical analyses were performed using Student's *t*-test, one-way analysis of variance (ANOVA) with Bonferroni's or Tukey's post hoc tests, or two-way ANOVA with Dunnet's post hoc test as appropriate using commercially available software (GraphPad Prism, GraphPad 15 Software). A *P*-value of <0.05 was considered statistically significant.

## RESULTS AND DISCUSSION

NANPs can be engineered to perform a variety of therapeutic and diagnostic functions due to the natural biological roles of RNA and DNA (1,2,11,15,31,32). However, the transition of NANPs to clinical use has been limited due to issues regarding production, chemical instability, intracellular delivery and off-target stimulation of the immune system (12,33,34). We have examined the characteristics of a panel of novel NANPs that may overcome many of these problems.

### NANP composition defines their physicochemical properties

The NANP panel used in these studies was designed to self-assemble in one-pot with high batch-to-batch consistency due to formation of either A-form or B-form helices *via* canonical Watson-Crick interactions (3), which has the potential to become cost effective and advantageous should future scaled-up production be warranted (15). We constructed seven triangular NANPs with differing compositions shown in Figure 1: all RNA (RcR), RNA center and DNA sides (RcD), RNA center and 2′F U/C modified RNA sides (Rc2′F), all DNA (DcD), DNA center and RNA sides (DcR), DNA center and 2′F U/C modified RNA sides (Dc2′F), and a fully 2′F U/C modified (2′Fc2′F) RNA triangles. We confirmed the intended assemblies of all NANPs using AFM imaging and native-PAGE (Figure 1B-C), and the diameter of the assembled NANPs was determined to be ∼14–17 nm (Figure 1D) by dynamic light scattering (DLS). These observed size differences can be attributed to nucleic acid helix formation given that RNA/RNA, 2′F/RNA and 2′F/DNA fold into an A-form helix while DNA/DNA folds into a B-form helix that is narrower and more elongated.

The thermodynamic stabilities of the NANPs were compared using the UV-melting technique and the data was graphed and fitted to determine the melting range of each NANP (Supporting Figure S1). As shown in Figure 1D, two distinct individual melting temperatures (*T*_m_) were seen for RcD, DcR, Rc2′F, Dc2′F and 2′Fc2′F NANPs, and one *T*_m_ for the RcR and DcD triangles. In agreement with our previous works, complete dissociation tended to occur at higher temperatures for NANPs with a central RNA strand (3,6,35). Consistent with previous findings, incorporation of 2′F modified strands resulted in higher *T*_m_s, with the fully modified NANPs displaying the highest *T*_m_ due to better stacking between adjacent base-pairs within the resulting secondary structure (36,37).

The clinical use of RNA NANPs has been limited because the presence of hydroxyl groups makes them susceptible to hydrolysis by nucleases and divalent metal ions (33). We therefore compared the serum stability of the NANP panel by agarose gel electrophoresis following incubation with FBS (Figure 1D). Interestingly, we have identified two composition modifications that increase NANP serum stability. We found that DcD triangles displayed greater serum stability compared to RcR, RcD, and DcR triangles as ribonucleases are more abundant than deoxyribonuclease in FBS. Additionally, the incorporation of 2′F U/C modified strands increased serum stability over a 12-h time period as shown by the Rc2′F, Dc2′F, 2′Fc2′F NANPs due to the reduction of hydroxyl groups that promote recognition by nucleases.

In the absence of a carrier, cells do not internalize our NANPs due to their charge (6). As such, we have assessed the structural integrity of these NANPs when combined with the transfection reagents L2K and DOTAP. We found all NANPs remained intact following incubation with either carrier and treatment with Triton X-100, used in our transfection experiments to aid NANP release, as determined by native-PAGE (Figure 1E and Supporting Figure S2). The observed differences in band intensity for RNA and DNA NANPs are attributed to the strong binding preference of ethidium bromide to double stranded DNA. Importantly, each NANP structure displays similar band intensities in the control and carrier liberated samples.

### Carrier and NANP composition affect internalization and subcellular localization

**Figure 2.**
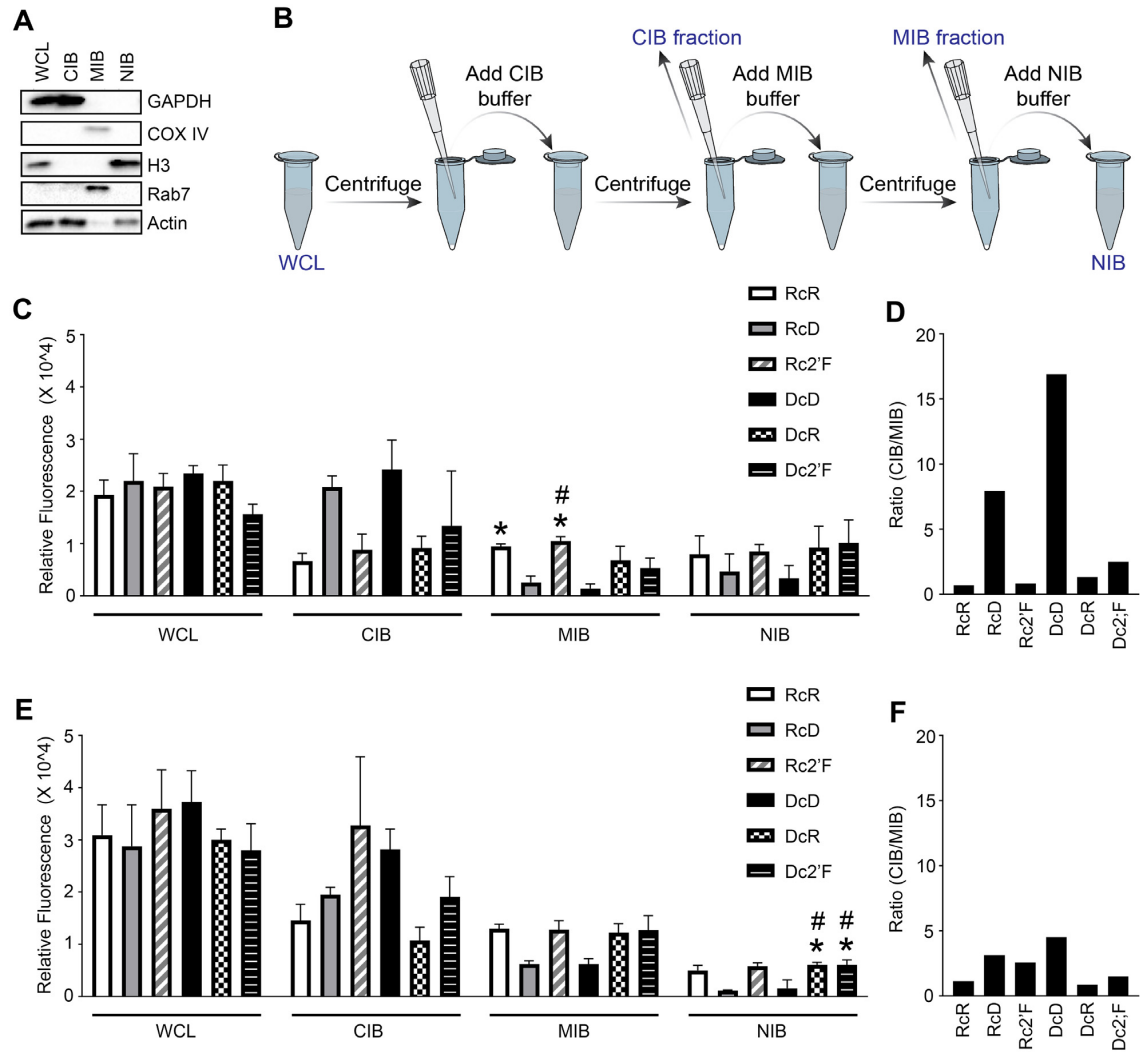
NANPs show enrichment in membrane bound compartments and cytosolic compartments. Microglia were transfected with 5 nM Cy3 labeled NANPs and at 2- and 4-h post transfection, whole cell lysates (WCL) were fractioned into cytosolic (Cytosolic Isolation Buffer, CIB), membrane/organelle (Membrane/organelle isolation buffer, MIB) and nuclear/cytoskeleton (Nuclear/cytoskeleton isolation buffer, NIB) fractions. (**A**) Fractions were evaluated for protein expression of GAPDH, COX IV, histone 3 (H3), Rab7, and Actin using immunoblot analysis. (**B**) Schematic of cell fractionation protocol. Fluorescence (Ex540/Em580) of cell fractions was evaluated using a SpectraMax iD5 plate reader from molecular devices at (**C**) 2 h and (**E**) 4 h. The DNA-Cy3 strand used to compose all DcD, DcR and Dc2′F is 1.74 ± 0.005 times less fluorescent than the RNA-Cy3 strand used to compose RcR, RcD, Rc2′F; therefore, the fluorescent signal of DcD, DcR, and Dc2′F in each fraction was multiplied by 1.74 to compensate for the lower fluorescent signal observed with the DNA-Cy3 strand. Data are represented as the mean ± (SEM) for a minimum of three independent experimental replicates. Asterisks indicate statistical significance compared to cells transfected with DcD NANPs and pound symbols indicate statistical significance compared to cells transfected with RcD NANPs (one-way ANOVA, *P*-value < 0.05). The ratio of the CIB fraction to the MIB fraction is displayed at (**D**) 2 h and (**F**) 4 h.

**Figure 3.**
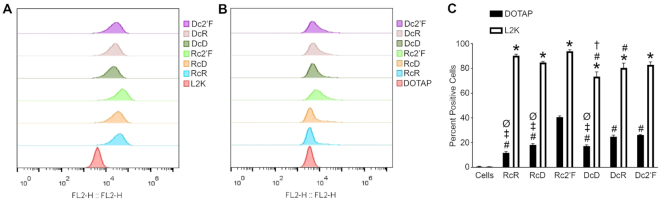
Relative cellular uptake of NANPs determined by flow cytometry. (**A**) Microglia were transfected with 5 nM Cy3-labeled NANPs using L2K for 4 h. (**B**) Microglia were transfected with 5 nM Cy3-labeled NANPs using DOTAP for 4 h. (**C**) The average percent positive cells for NANPs delivered with L2K and DOTAP. Data are represented as the mean ± (SEM) for a minimum of three independent experimental replicates. Asterisks indicate statistical significance for NANP delivery with DOTAP versus L2K. Pound symbols indicate statistical significance compared to Rc2′F NANPs delivered with the same carrier. Dagger symbols indicate statistical significance compared to RcR NANPs delivered with the same carrier. Double dagger symbols indicate statistical significance compared to DcR NANPs delivered with the same carrier. ᴓ indicate statistical significance compared to Dc2′F NANPs delivered with the same carrier (two-way ANOVA, *P*-value < 0.05).

Carrier selection can affect NANP delivery efficiency and so we have examined the internalization and subcellular trafficking of all NANPs when delivered with either of two polycationic lipid-based carriers. Microglia were transfected with Cy3 labeled NANPs for 2 and 4 h and whole cell lysates (WCL) were separated into three fractions: cytosolic, membrane/organelle, and nuclear/cytoskeleton. The purity of each fraction was determined by immunoblot analysis for the presence of the cytosolic, mitochondria/organelle, and nuclear/cytoskeleton protein markers, GAPDH, COX IV and Rab7 and histone 3, respectively (Figure 2A). The fluorescence of the WCL and each fraction was quantified as a measure of NANP enrichment at 2 and 4 h (Figure 2C and E, respectively). A similar fluorescence signal was observed in the WCL at 2 and 4 h for all NANPs indicating successful and comparable intracellular delivery of each. However, L2K was found to deliver NANPs more effectively than DOTAP (Figure 3) as determined by flow cytometry (73.4–93.8% versus 11.8–40.6% positive cells, respectively). Interestingly, we also observed differences in delivery efficiency with each carrier according to the composition of the NANP. Using L2K as a carrier, delivery efficiency decreased in the following NANP order from highest to lowest: Rc2′F, RcR, RcD, Dc2′F, DcR and DcD. A different trend was observed using DOTAP as the carrier, with delivery efficiency decreasing in the following NANP order from highest to lowest: Rc2′F, Dc2′F, DcR, RcD, DcD and RcR. While the relative stability of 2F’ modified NANPs in serum may contribute to their greater uptake efficiency, it is important to note that complexing NANPs with lipid-based carriers provides some protection against nuclease degradation for all particles. As such, these data indicate that certain NANP-carrier complexes are superior to others in regard to delivery efficiency, with the highest delivery efficiency observed for Rc2′F NANPs for both carriers, but the specific aspects of NANP-carrier interactions that yield higher delivery efficiencies remain to be defined.

**Figure 4.**
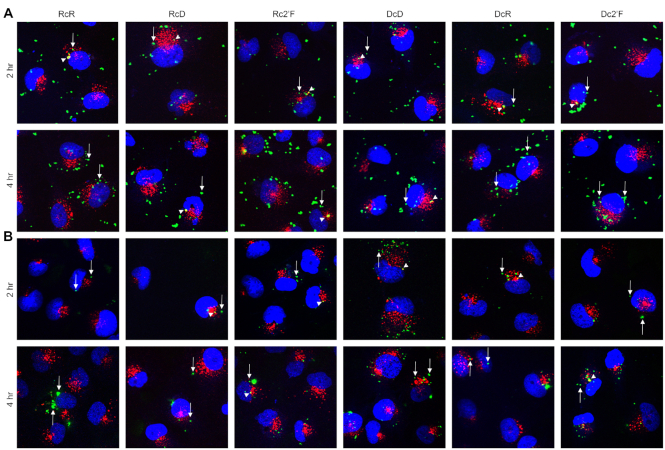
NANPs are internalized through endocytic pathway and traffic to the cytosol. Microglia were transfected with 5 nM Cy3-labeled NANPs using L2K (**A**) or DOTAP (**B**) for 2 or 4 h. Localization of NANPs (green) within cells was determined by immunofluorescence microscopy for the early endosomal marker, EEA1 (red), and nuclear stain DAPI (blue). Arrow heads indicate NANPs colocalized with EEA1, and arrows indicate NANPs that do not colocalize with EEA1.

For all NANPs, we observed fluorescence signals in both the membrane/organelle and the cytosolic fractions, suggesting that NANPs traffic through endosomal compartments to the cytosol. In agreement with this interpretation, immunofluorescence microscopy showed that some internalized NANPs colocalize with an early endosome marker while others do not, consistent with cytosolic localization (Figure 4). At 2 h, we observed marked differences in the enrichment of NANPs to the membrane/organelle fraction (Figure 2C), with the lowest fluorescence signals observed for the RcD and DcD NANPs. Importantly, we observed a statistically significant difference between the enrichment of DcD NANPs to the membrane/organelle fraction compared to both the RcR and Rc2′F NANPs. Additionally, there was a significant difference between the enrichment of RcD NANPs and the Rc2′F NANPs to the membrane/organelle fraction. While we did not observe a significant difference in the enrichment of NANPs to the cytosolic fraction at 2 h, it is important to note that the RcD and DcD NANPs generally displayed the highest cytosolic fluorescence signals.

Interestingly, when we examined the cytosolic to membrane/organelle fluorescence ratio at 2 h (Figure 2D) only the RcR and Rc2′F NANPs displayed a ratio <1, indicating preferential localization to the membrane/organelle fraction. In contrast, the RcD, DcD, DcR and Dc2′F NANPs all displayed a ratio >1, suggesting preferential localization to the cytosolic fraction, with the RcD and DcD NANPs having ratios of 7.9 and 16, respectively. At 4 h (Figure 2F) all NANPs, except the DcR, displayed a ratio above 1 (range 1.1–4.5) indicating that almost all the NANPs transitioned to the cytosolic compartment over time. We observed a decrease in the cytosolic to membrane/organelle fluorescence ratio for RcD and DcD NANPs from 2 to 4 h suggesting that, as more NANPs are internalized, there is a change in the rate of NANPs trafficking to the cytosolic compartment. Finally, we found at least some NANP enrichment to the nuclear fraction at both 2 and 4 h (Figure 2C and E) with the lowest fluorescence signal for the RcD and DcD NANPs, similar to that seen in the membrane/organelle fraction. RcD and DcD NANPs displayed significantly reduced enrichment to the nuclear/cytoskeleton fraction at 4 h compared to both the DcR and Dc2′F NANPs (Figure 2E).

Once again, we noted that each NANP composition displayed different delivery efficiencies with the same carrier. When L2K was the carrier, Rc2′F, followed by RcR, was delivered the most efficiently. When DOTAP was the carrier, Rc2′F was again the most efficiently delivered but this was followed by DcR and Dc2′F. As such, these data suggest that the carrier affects delivery efficiency and that certain carrier-NANP combinations display greater delivery efficiency than others. It is important to note that, while we examined two lipid-based carriers in the present study, other carriers such as polymers, silicon and carbon materials (9,10,38) that differ in their mechanism and efficiency of delivery might also be employed, and future studies will be required to explore their ability to deliver these NANPs and their intracellular trafficking.

### Composition affects NANP-induced cytokine production mediated by NF-κB and IRF

**Figure 5.**
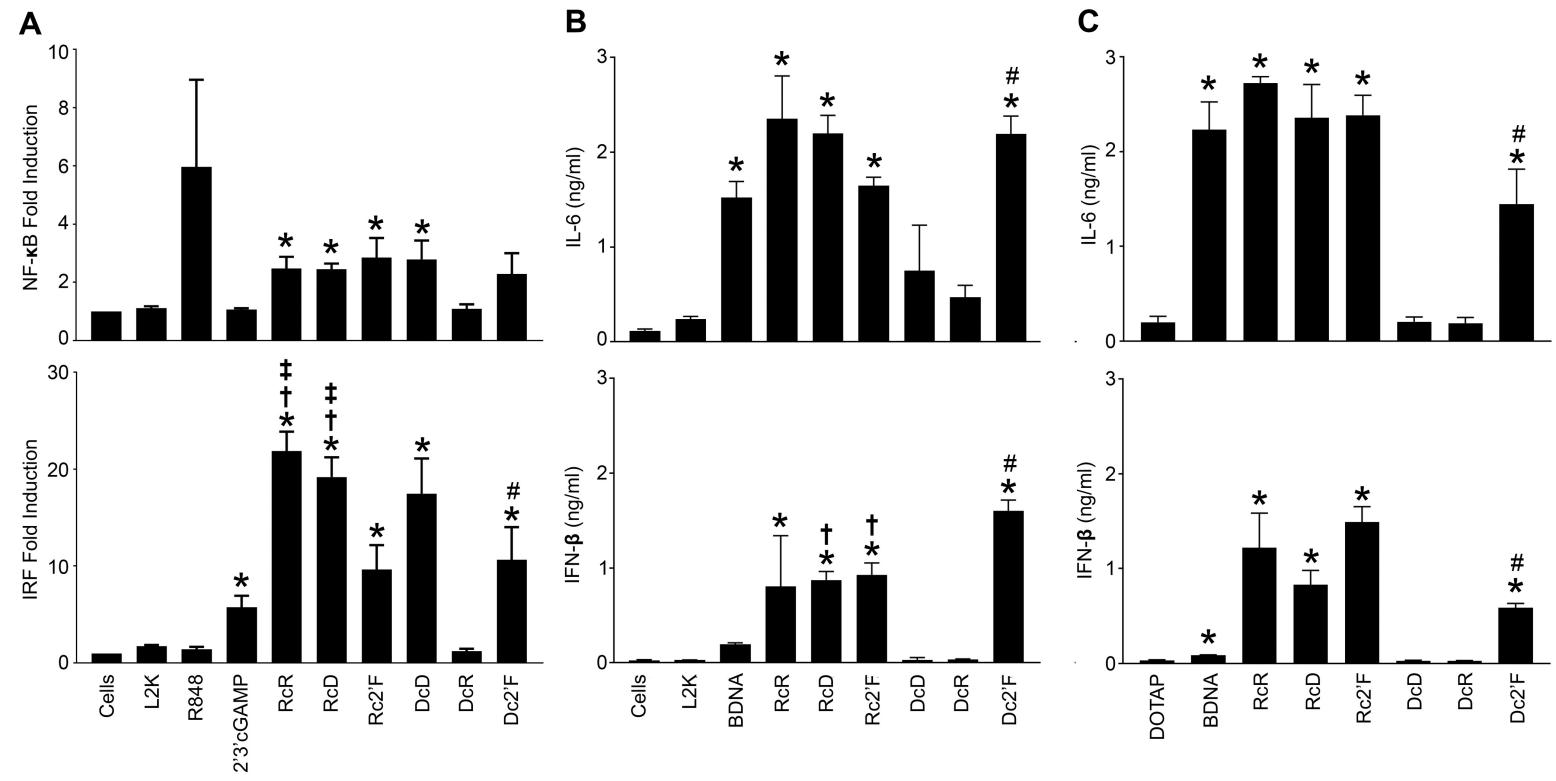
2′F modified NANPs stimulate NF-κB and IRF mediated cytokine production. (**A**) THP-1 Dual reporter cells were transfected with 5 nM NANPs for 24 h using L2K. Induction of the NF-κB pathways was conducted using Quanti-Blue to monitor SEAP activity. Induction of the IRF pathway was conducted using Quanti-Luc to monitor the activity of secreted luciferase. (B, C) Microglia were transfected with 5 nM NANPs using either L2K (**B**) or DOTAP (**C**) for 4 h and cell supernatants were collected 24 h post transfection. Cell supernatants were then analyzed for cytokine production using specific capture ELISAs for IL-6 and IFN-β. Data are represented as the mean ± (SEM) for a minimum of three independent experimental replicates. Asterisks indicate statistical significance compared to L2K, pound symbols indicate statistical significance compared to DcR, dagger symbols indicate statistical significance compared to Dc2′F, double dagger symbols indicate statistical significance compared to Rc2′F (Student's *t*-test, *P*-value < 0.05).

We examined the immunostimulatory activity of the NANP panel using THP-1 dual reporter cells possessing inducible reporter constructs to monitor NF-κB and IRF pathway activation. As shown in Figure 5A, RcR and RcD NANPs stimulated NF-κB and IRF signaling pathways, as did Rc2′F NANPs. We also observed, DcD and Dc2′F NANPs stimulated both these signaling pathways (Figure 5A) but DcR NANPs did not have a significant effect on either NF-κB or IRF signaling (Figure 5A).

Activation of NF-κB and IRF signaling pathways leads to the production of proinflammatory cytokines such as IL-6 and type I IFNs such as IFN-β, respectively. Therefore, we next examined IL-6 and IFN-β production in response to NANPs delivered with either L2K (Figure 5B) or DOTAP (Figure 5C). Consistent with findings obtained with the reporter cell lines, we found that with RcR and RcD NANPs delivered with either carrier stimulated significant IL-6 and IFN-β production by microglia while DcR NANPS did not (Figure 5B and C). Interestingly, DcD also failed to induce significant IL-6 and IFN-β release (Figure 5B and C) despite an ability to activate NF-κB and IRF signaling (Figure 5A). Taken together, this data indicates that the nucleic acid composition of NANPs can be altered to direct inflammatory cytokine and IFN responses. This interpretation is further supported by our analysis of the immunostimulatory properties of all 16 possible RNA and DNA combinations for triangle NANPs, in which we observed a gradual shift in the ability to induce cytokine production as the composition is changed strand-by-strand from RNA to DNA (Supporting Figure S3). In the present study, we have focused exclusively on the effect of nucleic acid composition on the immunostimulatory activity of triangular NANPs, but it should be noted that we have previously demonstrated that the connectivities and sizes of 2D polygons and 3D shapes can also contribute to such differences (6).

In addition, we examined the ability of 2′F U/C modification to modulate NANP immunoreactivity and we determined that Rc2′F NANPs stimulate IL-6 and IFN-β production in a similar manner to RcR and RcD NANPs indicating that this modification does not impact immune mediator responses to RNA NANPs. Interestingly, we noted that Dc2′F NANPs can stimulate NF-κB and IRF activation and elicit IL-6 and IFN-β production, in contrast to DcR and DcD NANPs, indicating that 2′F modification significantly alters the immunoreactivity of DNA NANPs. Finally, we examined the immunoreactivity of a fully modified 2′Fc2′F NANP and found that these NANPs can stimulate significant IL-6 and IFN-β production in a similar manner to Rc2′F and Dc2′F NANPs (Supporting Figure S4).

### NANP composition affects PRR activation

**Figure 6.**
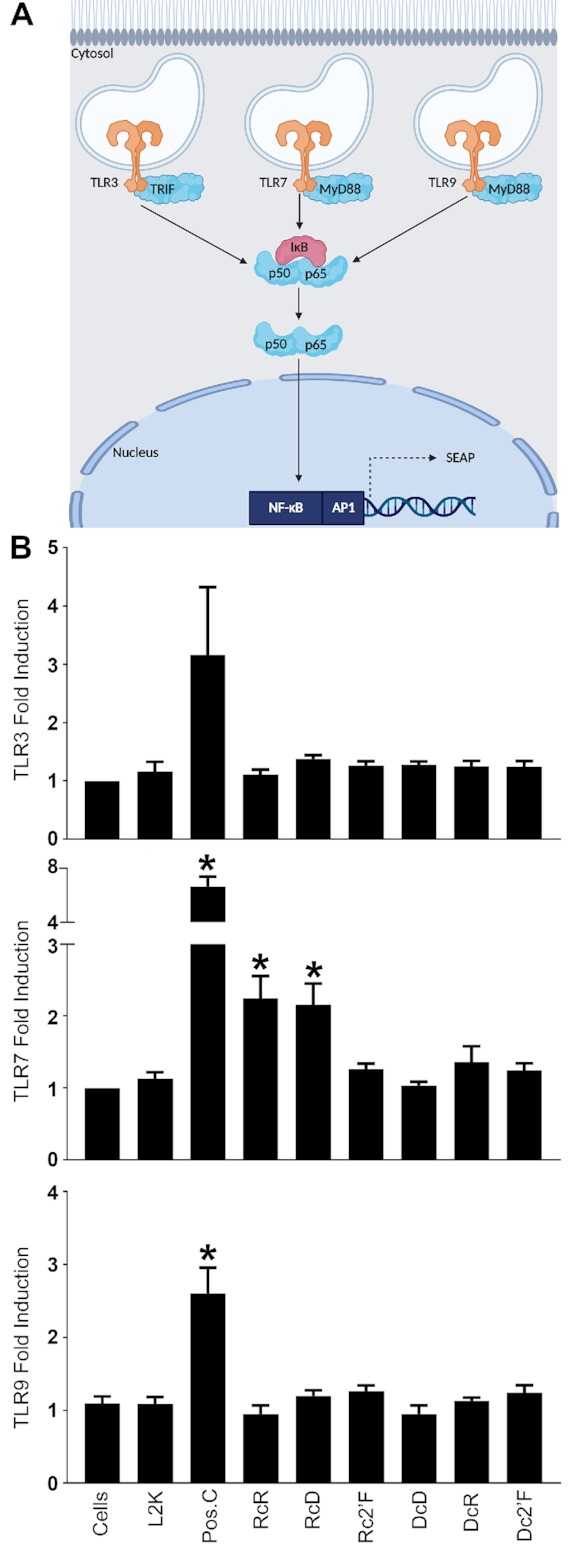
RcR and RcD NANPs stimulate TLR7 responses. (**A**) Schematic of HEK-Blue TLR reporter cells. Created with Biorender.com (**B**) HEK-Blue TLR reporter cells were transfected with 5 nM NANPs for 24 h. TLR induced NF-κB activity was evaluated using Quanti-Blue to monitor SEAP activity. Poly (I:C), R848 and ODN 2006 were used as positive controls for TLR3, TLR7 and TLR9, respectively. Data are represented as the mean ± (SEM) for three independent experimental replicates. Asterisks indicate statistical significance compared to L2K (Student's *t*-test, *P*-value < 0.05).

Nucleic acid PRRs are localized to specific subcellular compartments and can identify specific nucleic acid signature motifs. Given the observation that NANP composition affects subcellular localization, we next examined NANP detection by nucleic acid sensors localized in both the endosomal and cytosolic compartments. Our data indicates that NANPs reside, or traffic through, membrane/organelle fractions. The endosome contains three TLRs, TLR3, TLR7 and TLR9, that are known to recognize double stranded RNA, single stranded or double stranded RNA, and unmethylated CpG DNA, respectively (39,40). We used HEK-Blue reporter cell lines that express either human TLR3, TLR7 or TLR9 to determine if NANPs initiate NF-κB activation via these endosomal TLRs. Surprisingly, while specific agonists for TLR3, TLR7 and TLR9 (poly (I:C), R848, and ODN 2006, respectively), induced NF-κB signaling in their corresponding reporter cell lines, none of these NANPs stimulated TLR3 or TLR9-mediated responses. The NANPs described here possess dsRNA containing 22 bp per side with stretches of single-stranded uracils at each corner, which may not be sufficient for TLR3 recognition as TLR3 requires ligands with greater than 40 bp (41). However, TLR7 can effectively recognize short dsRNAs as well as ssRNA (6,42,43). Consistent with this, we observed RcR and RcD NANPs were able to elicit TLR7-mediated NF-kB activation (Figure 6). Interestingly, Rc2′F NANPs failed to elicit such responses indicating that 2′F modification abrogates the ability of these NANPs to stimulate TLR7. This is consistent with previous studies that demonstrate replacement of the 2′ hydroxyl groups on single-stranded RNA abrogates TLR7 activation (26,44).

**Figure 7.**
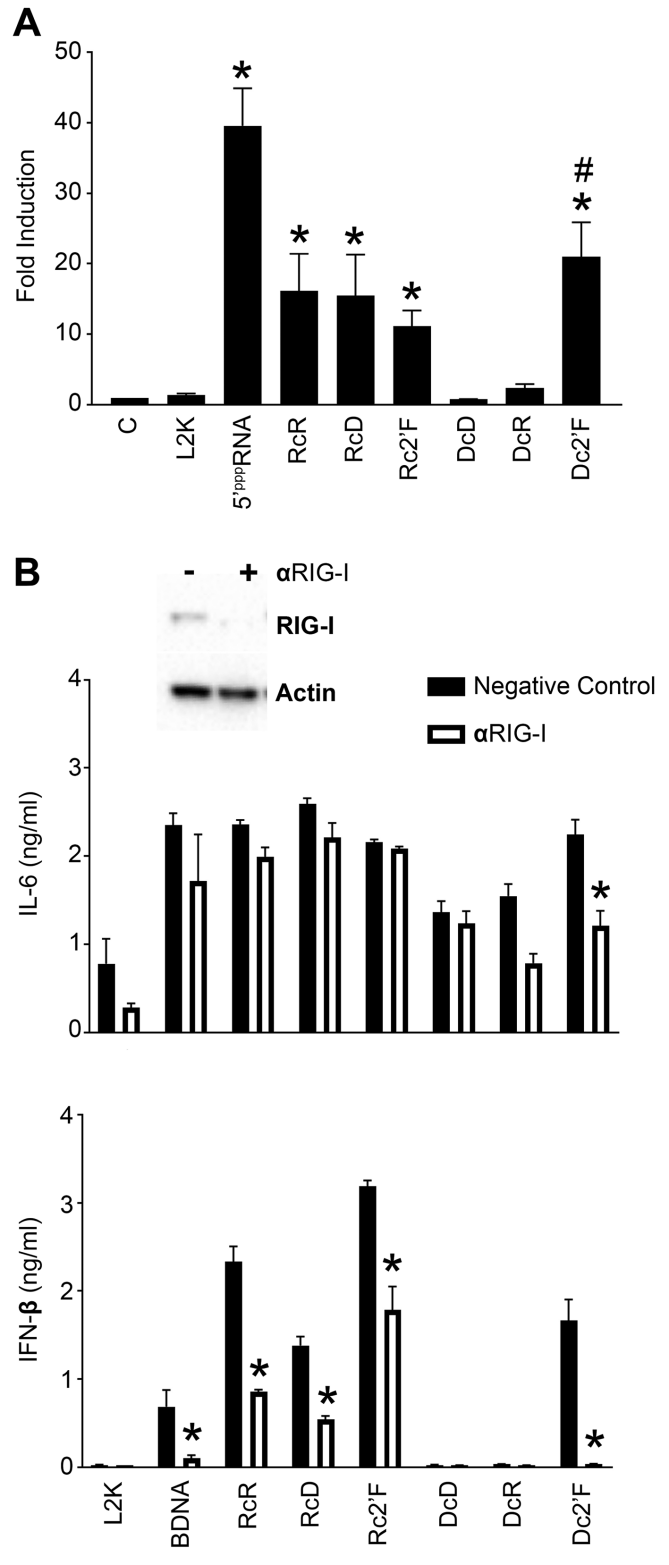
RcR, RcD, Rc2′F and Dc2′F NANPs stimulate RIG-I dependent responses. (**A**) HEK-Lucia RIG-I reporter cells were transfected with 5 nM NANPs for 24 h. RIG-I activity was evaluated using Quanti-Luc to monitor luciferase activity. (**B**) Microglia were treated with scrambled siRNA or siRNA targeting RIG-I (αRIG-I) at a final concentration of 5 nM for 24 h. Cells were placed in fresh media for 24 h prior to transfection with 5 nM NANPs. Cell supernatants and lysates were collected 24 h post transfection. Cell lysates were evaluated for RIG-I protein expression via immunoblot analysis. Cell supernatants were evaluated for IL-6 and IFN-β by specific capture ELISAs. Data are represented as the mean ± (SEM) for a minimum of three independent experimental replicates. Asterisks indicate statistical significance compared to L2K (A) or compared to the corresponding negative control abbreviated as C. (Student's *t*-test or two-way ANOVA, *P*-value < 0.05, DcR *P*-value = 0.0507).

We also observed NANPs enrich to the cytosolic fraction and there are several RNA and DNA sensors that are known to survey the cytosol for nucleic acids (45,46). Importantly, we have previously demonstrated that NANPs containing a central RNA strand are potent inducers of IFN-β (3,18,21). While both MDA-5 and RIG-I serve as cytosolic RNA sensors, our NANPs consist of relatively short nucleic acid strands that do not meet the minimum base pair requirement to function as MDA-5 ligands (47,48). Accordingly, we examined the role of RIG-I in NANP detection and found that RcR, RcD and Rc2′F RNA-based NANPs trigger responses in an HEK293 RIG-I reporter cell line (Figure 7A). In contrast, DNA-based NANPs, DcD and DcR, failed to elicit demonstrable RIG-I responses (Figure 7A).

In order to confirm the importance of RIG-I in the detection of our NANPs, we utilized siRNA to knock down RIG-I expression in microglia (Figure 7B) prior to NANP transfection with L2K (Figure 7) or DOTAP (Supplemental Figure S5). In agreement with our results in the RIG-I reporter cell line, RIG-I knockdown significantly diminished IFN-β release, but not IL-6 production, in response to RcR, RcD and Rc2′F modified, NANPs (Figure 7B and Supplemental Figure S5). RIG-I is a known receptor for 5′ triphosphorylated RNA ligands (47). As such, it was not surprising that RcR, RcD and Rc2′F, NANPs triggered RIG-I mediated responses as their central RNA strands possess 5′ triphosphates as a result of their transcriptional generation. In support of this, DcR NANPs that were generated from synthetic RNA strands lacking 5′ triphosphates failed to elicit RIG-I dependent responses.

**Figure 8.**
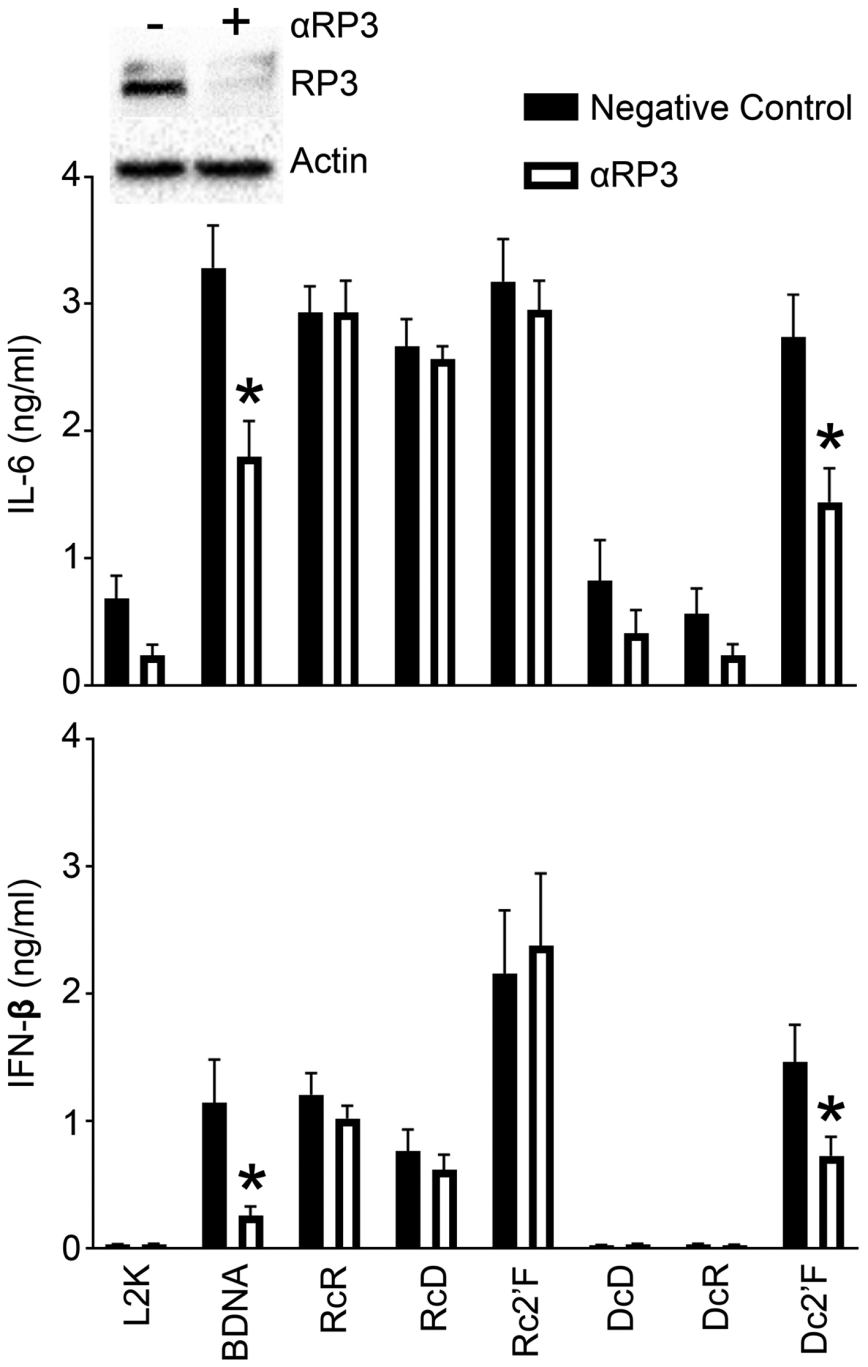
Dc2′F NANPs stimulate RNA polymerase III dependent responses. Microglia were treated with scrambled siRNA or siRNA targeting RNA polymerase III subunit A (αRP3) at a final concentration of 5 nM for 24 h. Cells were placed in fresh media for 24 h prior to transfection with 5 nM NANPs. Cell supernatants and lysates were collected 24 h post transfection. Cell lysates were evaluated for RNA polymerase III subunit A protein expression via immunoblot analysis (A). Cell supernatants were evaluated for IL-6 and IFN-β by specific capture ELISAs. Data are presented as the mean ± (SEM) for a minimum of three independent experimental replicates. Asterisks indicate statistical significance compared to the corresponding negative control. (two-way ANOVA, *P*-value < 0.05).

Interestingly, RIG-I has previously been shown to respond to DNA ligands indirectly due to RNA polymerase III-dependent transcription of AT-rich dsDNA (49,50). Indeed, we show that Dc2′F NANPs stimulate RIG-I activation using a RIG-I reporter cell line (Figure 7A) and Dc2′F NANP-mediated IFN-β and IL-6 production was significantly reduced following RIG-I knockdown (Figure 7B), indicating that this DNA-based NANP triggers RIG-I dependent responses. In order to determine if RNA polymerase III (Pol III) is required for Dc2′F NANP identification, we utilized siRNA to knock down the expression of the catalytic subunit of RNA polymerase III (Pol III subunit A) in microglia prior to NANP transfection with L2K (Figure 8) or DOTAP (Supporting Figure S5). As shown in Figure 8, Pol III subunit A knockdown significantly reduced IL-6 and IFN-β responses to BDNA, a ligand known to be recognized by RIG-I in a Pol III-dependent manner (30,49). Importantly, Pol III subunit A knockdown significantly reduced Dc2′F NANP-induced IL-6 and IFN-β release indicating that this NANP is recognized by RIG-I in a Pol III-dependent manner (Figure 8). Notably, altering the sequences of Dc2′F NANP strands may affect the efficiency of RNA polymerase III mediated transcription and subsequent immune activation due to the preferential binding of RNA polymerase III to AT-rich DNA ligands.

## CONCLUSION

**Figure 9.**
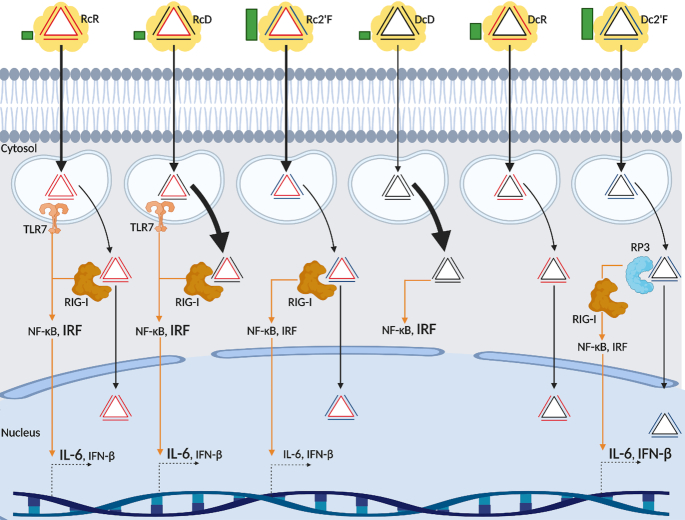
NANP trafficking and immunostimulation. Green bars indicate levels of serum stability. Black arrows indicate NANP trafficking and localization. Yellow cloud represents the carrier. All NANPs are successfully delivered to cells using L2K and transition from an endosomal compartment to the cytosol. Thickness of black arrows indicates degree of nanoparticle internalization and accumulation. Rc2′F and RcR displayed the highest degree of internalization and DcD displays the lowest degree of internalization. RcD and DcD more rapidly accumulate in the cytosol. We observed RcR, Rc2'F, DcR and Dc2'F display localization in the nuclear fraction. Orange arrows indicate immunostimulation and signaling. Within the endosomal compartment RcR and RcD activate TLR7. Once in the cytosol, RcR, RcD, and Rc2'F activate RIG-I directly. Dc2'F is converted by RNA polymerase III to an RNA intermediate which can activate RIG-I. TLR7 and RIG-I signaling cascades trigger NF-κB and IRF activation and production of IL-6 and IFN-β. Font size indicates the degree of NF-κB and IRF activation or the degree of IL-6 and IFN-β production. Created with BioRender.com

Taken together, our data indicates that NANP composition affects subcellular trafficking independent of the carrier molecule as summarized in Figure 9. While all the NANPs studied were internalized and trafficked through a membrane bound compartment to the cytosol, RcD and DcD NANPs rapidly and preferentially localize to the cytosol with minimal enrichment to the membrane/organelle and the nuclear/cytoskeleton regions. In contrast, RcR, Rc2′F, DcR and Dc2′F NANPs preferentially localize to the membrane/organelle regions at early time points and proceed to the cytosol at later time points. Additionally, these NANPs display some trafficking to the nucleus/cytoskeleton. This suggests that NANP composition could be engineered to deliver therapeutic and/or diagnostic tools to specific subcellular compartments. While in the present study we have examined a small panel of triangular NANPs with lipid-based carriers, NANPs can be assembled with an almost limitless number of conformations comprised of RNA, DNA, and chemically modified strands, and can be delivered with a wide variety of carriers (3,9,10,21,38,51–55). This provides a potentially large library of NANP-carrier combinations that could be developed for specific clinical application.

In addition, we demonstrated that NANP composition may be engineered to avoid off-target detrimental inflammation or even to enhance desirable immune responses (Figure 9). Off-target stimulation of the immune system can result in potentially lethal systemic inflammation and has remained a major hurdle for the clinical application of NANPs. We have previously modeled NANP quantitative structure-activity relationships and demonstrated that the physicochemical properties of NANPs, which are defined by nucleic acid composition, are strong predictors of immunostimulatory potential (21). In agreement with our previous data, we demonstrated that NANP composition can be modified to limit immune mediator production. We showed that DcD and DcR DNA-based NANPs failed to elicit significant IL-6 and IFN-β production and DNA strand incorporation in our comprehensive triangle panel, in general, reduced cytokine responses. In contrast, RNA NANPs promoted significant IFN-β production, and incorporation of a 2′F modified strand in DNA NANPs resulted in a potent ability to induce release of this cytokine.

Nucleic acid sensing PRRs are strategically located in the endosomal and the cytosolic compartments to screen for foreign components and host cell damage. Activation of these receptors is known to stimulate IFN-β production that can promote protective antiviral responses and antigen presentation to trigger adaptive immune responses. As such, PRR agonists show great promise as antiviral agents and vaccine adjuvants. The present study indicates that NANP modification can be used to either promote or avoid PRR activation. For example, we have shown that RcR and RcD NANPs function as TLR7 agonists and such agents, including the FDA approved TLR7/8 agonist imiquimod, are being explored for use as antivirals, cancer therapeutics and vaccine adjuvants (56–58) (59,60). Importantly, we demonstrate that altering the RNA to DNA ratio of NANPs can be used as a means of control off-target immunostimulation, and we have found that TLR7 activation can be avoided by using DcD, DcR, and Dc2′F based NANPs. Furthermore, we show that TLR7 activation can also be abrogated by chemical modification as evidenced by the inability of R2′F modified RNA NANPs to stimulate TLR7 responses.

Similarly, RIG-I ligands are also emerging as promising antiviral agents and vaccine adjuvants. Currently, many antivirals are direct-acting agents that are virus specific and can be rendered ineffective as viruses evolve evasion mechanisms. RIG-I agonists including 5′ tri- and di-phosphorylated short dsRNA show promise as pan-antiviral agents because they promote the expression of IFN-stimulated genes and other antiviral products (61). RIG-I agonists have been shown to provide protection in murine models of influenza virus and Dengue virus infection (61,62), and 5′ triphosphorylated dsRNAs have been shown to promote greater increases in specific antibody titers elicited by influenza virus like-particles or influenza vaccine administration than currently approved adjuvants (63,64). However, despite the prospects for RNA-based therapeutics, their utilization is currently limited by our inability to directly deliver these agents into cells in the absence of a carrier and by their enzymatic instability. In the present study, we demonstrate that NANPs can be successfully delivered with lipid-based carriers and that four NANPs, RcR, RcD, Rc2′F and Dc2′F, stimulate RIG-I dependent responses. RcR, RcD and Rc2′F, all directly stimulate RIG-I responses due to the presence of 5’triphosphates on the central RNA strand, while Dc2′F, but not DcR, NANPs stimulate RIG-I dependent responses *via* a Pol III dependent mechanism. However, we cannot rule out that cytosolic DNA sensors may also contribute to the identification of Dc2′F NANPs. Collectively, this data indicates that the present NANP platform allows for the incorporation of DNA and chemically modified strands to increase serum stability while maintaining RIG-I agonist activity.

As such, this study provides evidence that NANP composition can be engineered to control thermodynamic and enzymatic stability, and describes a means to rationally design NANPs for therapeutic and/or diagnostic applications. These NANPs can be delivered with lipid-based carriers and the modification of their composition provides a mechanism to achieve targeted subcellular delivery. Furthermore, the composition of these NANPs can be designed to specifically avoid or induce innate immune sensor activation that can, in turn, either limit detrimental inflammation or enhance beneficial immune responses.

## DATA AVAILABILITY

The authors confirm that the data supporting the findings of this study are available within the article and/or its supplementary materials. Any additional data that support the findings of this study are available from the corresponding author, K.A. Afonin, upon reasonable request.

## Supplementary Material

gkaa908_Supplemental_File
