# Supplementary material for: The immunorecognition, subcellular compartmentalization, and physicochemical properties of nucleic acid nanoparticles can be controlled by composition modification
Source: Nucleic Acids Res. 2020 Oct 22;48(20):11785–98. doi: 10.1093/nar/gkaa908 (PMC7672449; doi:10.1093/nar/gkaa908)
Supplement: gkaa908_Supplemental_File [file gkaa908_supplemental_file.docx]

**SUPPORTING INFORMATION**

**The immunorecognition, subcellular compartmentalization, and physicochemical properties of nucleic acid nanoparticles can be controlled by composition modification**

M. Brittany Johnson^1^, Justin R. Halman^2^, Daniel K. Miller^3^, Joseph S. Cooper^3^, Emil F. Khisamutdinov^3^, Ian Marriott^1^, and Kirill A. Afonin^2*^

^1^Department of Biological Sciences, University of North Carolina at Charlotte, Charlotte, NC, USA; ^2^Nanoscale Science Program, Department of Chemistry, University of North Carolina at Charlotte, Charlotte, NC, 28223, USA; ^3^Department of Chemistry, Ball State University, Muncie, IN, 47306, USA.

Address correspondence to [kafonin@uncc.edu](mailto:kafonin@uncc.edu)

**NANPs used in this project**

| Name | sequence 5’ → 3’ |
| --- | --- |
| **All RNA triangle (RcR)** | |
| center | GGAUGCUGGUACUUUUGAAACAUUUCGAGUCGCGAGGGUUUUCCCAUCGUUGGCCCGUAUCGCGUUUUCUUAUGAAGA |
| Side 1 | GGUCGCGACCUUCUUUUCCCUCGCGACUCGAAAUGUUUCUUUUCGAGGUCGCCC |
| Side 2 | GGAUCUUUCGCCUUUUCGCGAUACGGGCCAACGAUGGGUUUUGAAGGUCGCGAC |
| Side 3 | GGGCGACCUCGUUUUGUACCAGCAUCCUCUUCAUAAGUUUUGGCGAAAGAUCC |
| **All DNA triangle (DcD)** | |
| center | GGATGCTGGTACTTTTGAAACATTTCGAGTCGCGAGGGTTTTCCCATCGTTGGCCCGTATCGCGTTTTCTTATGAAGA |
| Side 1 | GGTCGCGACCTTCTTTTCCCTCGCGACTCGAAATGTTTCTTTTCGAGGTCGCCC |
| Side 2 | GGATCTTTCGCCTTTTCGCGATACGGGCCAACGATGGGTTTTGAAGGTCGCGAC |
| Side 3 | GGGCGACCTCGTTTTGTACCAGCATCCTCTTCATAAGTTTTGGCGAAAGATCC |
| **All 2’F triangle* (2’Fc2’F)** | |
| center | GGAUGCUGGUACUUUUGAAACAUUUCGAGUCGCGAGGGUUUUCCCAUCGUUGGCCCGUAUCGCGUUUUCUUAUGAAGA |
| Side 1 | GGUCGCGACCUUCUUUUCCCUCGCGACUCGAAAUGUUUCUUUUCGAGGUCGCCC |
| Side 2 | GGAUCUUUCGCCUUUUCGCGAUACGGGCCAACGAUGGGUUUUGAAGGUCGCGAC |
| Side 3 | GGGCGACCUCGUUUUGUACCAGCAUCCUCUUCAUAAGUUUUGGCGAAAGAUCC |
| **DcR triangle** | |
| center | GGATGCTGGTACTTTTGAAACATTTCGAGTCGCGAGGGTTTTCCCATCGTTGGCCCGTATCGCGTTTTCTTATGAAGA |
| Side 1 | GGUCGCGACCUUCUUUUCCCUCGCGACUCGAAAUGUUUCUUUUCGAGGUCGCCC |
| Side 2 | GGAUCUUUCGCCUUUUCGCGAUACGGGCCAACGAUGGGUUUUGAAGGUCGCGAC |
| Side 3 | GGGCGACCUCGUUUUGUACCAGCAUCCUCUUCAUAAGUUUUGGCGAAAGAUCC |
| **RcD triangle** | |
| center | GGAUGCUGGUACUUUUGAAACAUUUCGAGUCGCGAGGGUUUUCCCAUCGUUGGCCCGUAUCGCGUUUUCUUAUGAAGA |
| Side 1 | GGTCGCGACCTTCTTTTCCCTCGCGACTCGAAATGTTTCTTTTCGAGGTCGCCC |
| Side 2 | GGATCTTTCGCCTTTTCGCGATACGGGCCAACGATGGGTTTTGAAGGTCGCGAC |
| Side 3 | GGGCGACCTCGTTTTGTACCAGCATCCTCTTCATAAGTTTTGGCGAAAGATCC |
| **Dc2’F triangle** | |
| center | GGATGCTGGTACTTTTGAAACATTTCGAGTCGCGAGGGTTTTCCCATCGTTGGCCCGTATCGCGTTTTCTTATGAAGA |
| Side 1 | GGUCGCGACCUUCUUUUCCCUCGCGACUCGAAAUGUUUCUUUUCGAGGUCGCCC |
| Side 2 | GGAUCUUUCGCCUUUUCGCGAUACGGGCCAACGAUGGGUUUUGAAGGUCGCGAC |
| Side 3 | GGGCGACCUCGUUUUGUACCAGCAUCCUCUUCAUAAGUUUUGGCGAAAGAUCC |
| **Rc2’F triangle** | |
| center | GGAUGCUGGUACUUUUGAAACAUUUCGAGUCGCGAGGGUUUUCCCAUCGUUGGCCCGUAUCGCGUUUUCUUAUGAAGA |
| Side 1 | GGUCGCGACCUUCUUUUCCCUCGCGACUCGAAAUGUUUCUUUUCGAGGUCGCCC |
| Side 2 | GGAUCUUUCGCCUUUUCGCGAUACGGGCCAACGAUGGGUUUUGAAGGUCGCGAC |
| Side 3 | GGGCGACCUCGUUUUGUACCAGCAUCCUCUUCAUAAGUUUUGGCGAAAGAUCC |

* Chemically modified RNA sequences contain only 2’ fluoro modified pyrimidines (U/C)

**Supporting Figures**

**
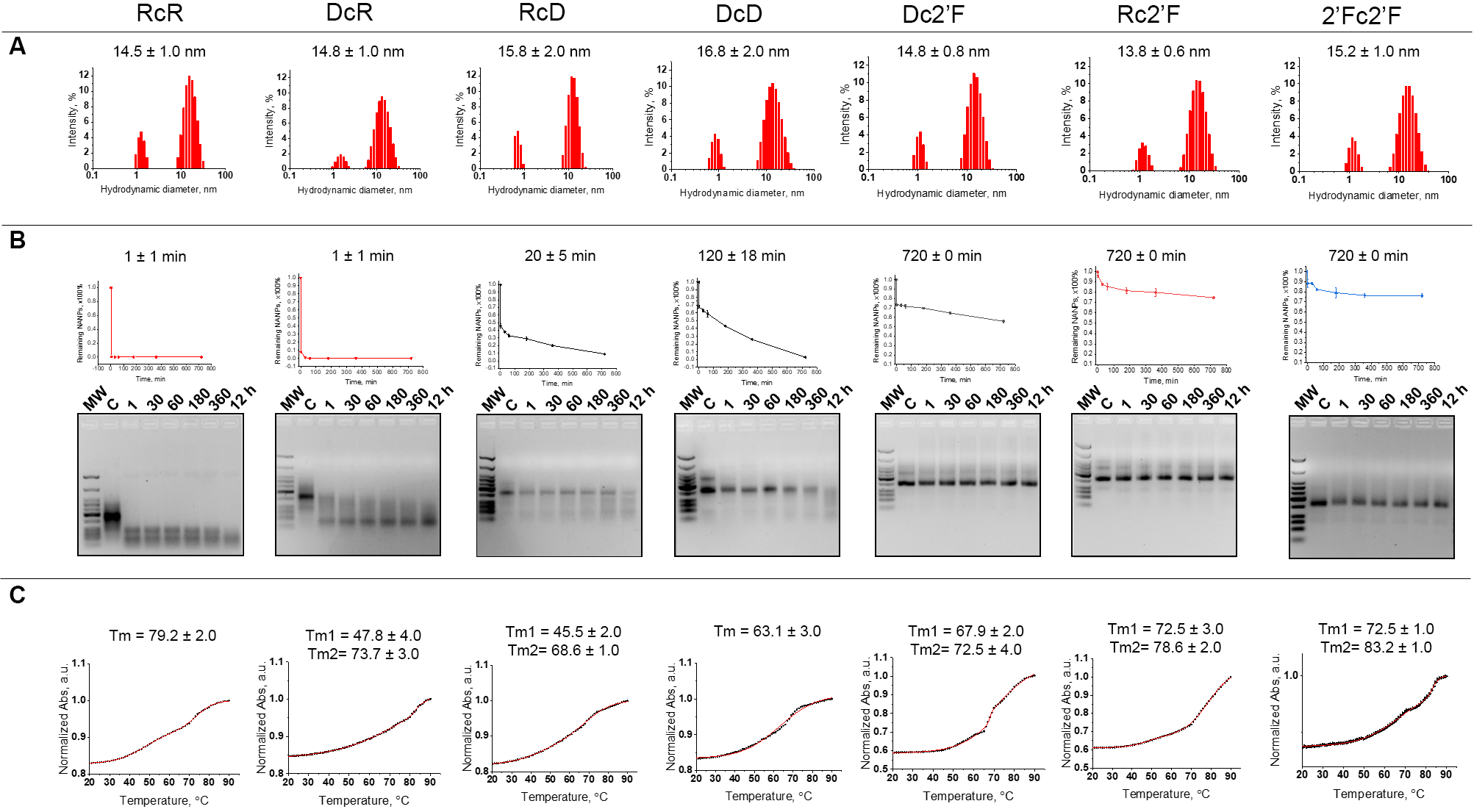
**

**Supporting Figure S1:** NANPs physicochemical characterization. Relative sizes of NANPs assessed by DLS (**A**), chemical stability of NANPs incubated in 20% FBS solution at 37°C from 1 - 720 minutes analyzed by EMSA (**B**), and melting temperatures assessed by UV-melting experiment (**C**).


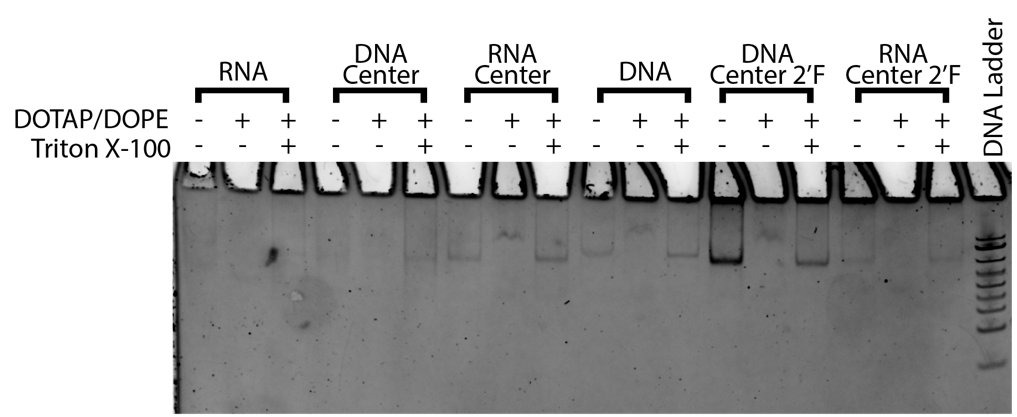


**Supporting Figure S2:** Structural integrity of NANPs upon release from DOTAP complexation confirmed by EtBr total staining native-PAGE.


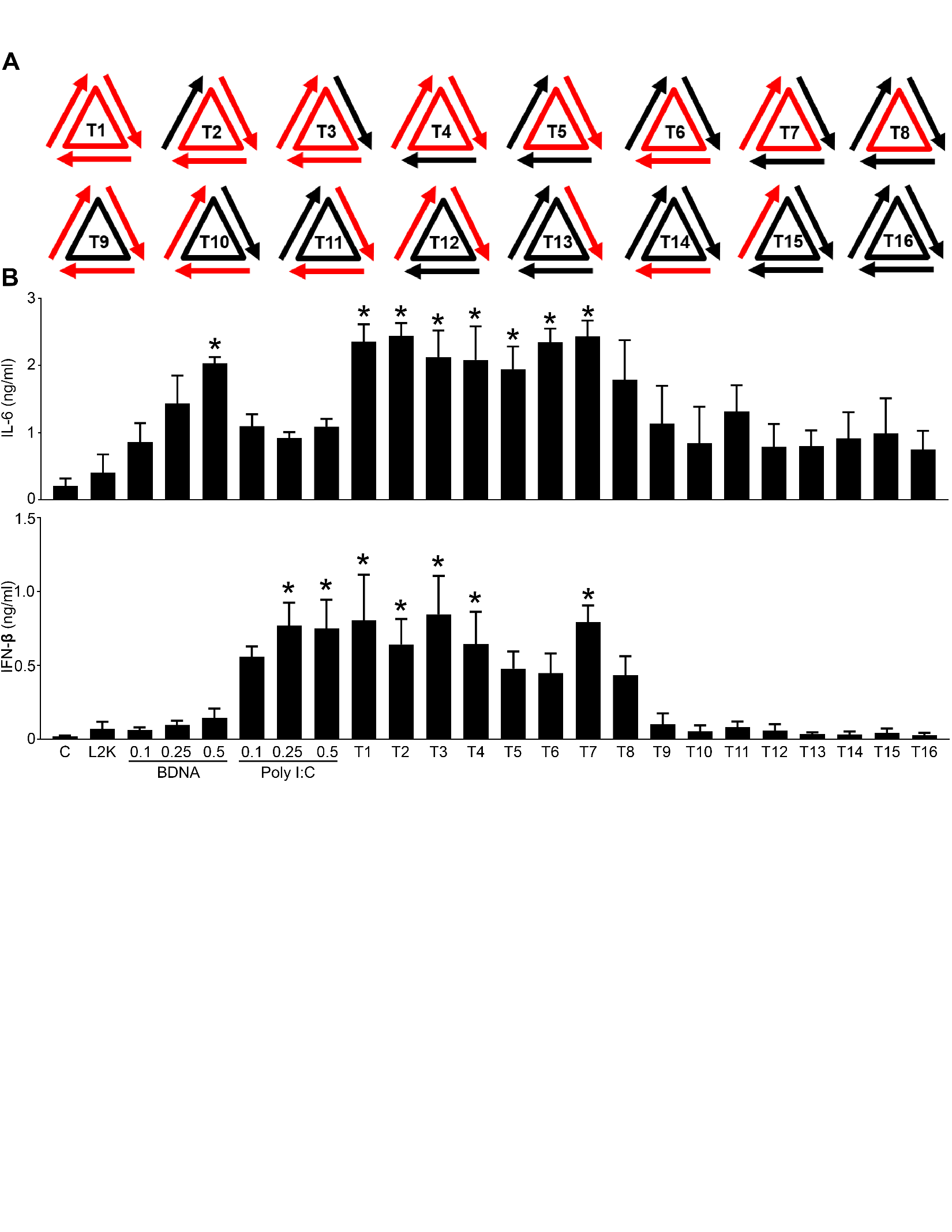
 **Supporting Figure S3.** Immunostimulation of NANP triangles measured via ELISAs for IL-6 and IFN-β. (**A**) Schematic of NANP triangles 1-16. RNA strands are colored in red and DNA is in black. (**B**) Microglia cells were transfected with 5 nM NANPs for 4 hours and cell supernatants were collected 24 hours post transfection and analyzed for cytokine production using specific capture ELISAs for IL-6 and IFN-β. Data are represented as standard error of the mean (SEM) for a minimum three independent experimental replicates. Asterisks indicate statistical significance compared to L2K (One-way ANOVA with Dunnett’s post hoc test, p-value < 0.05).

**
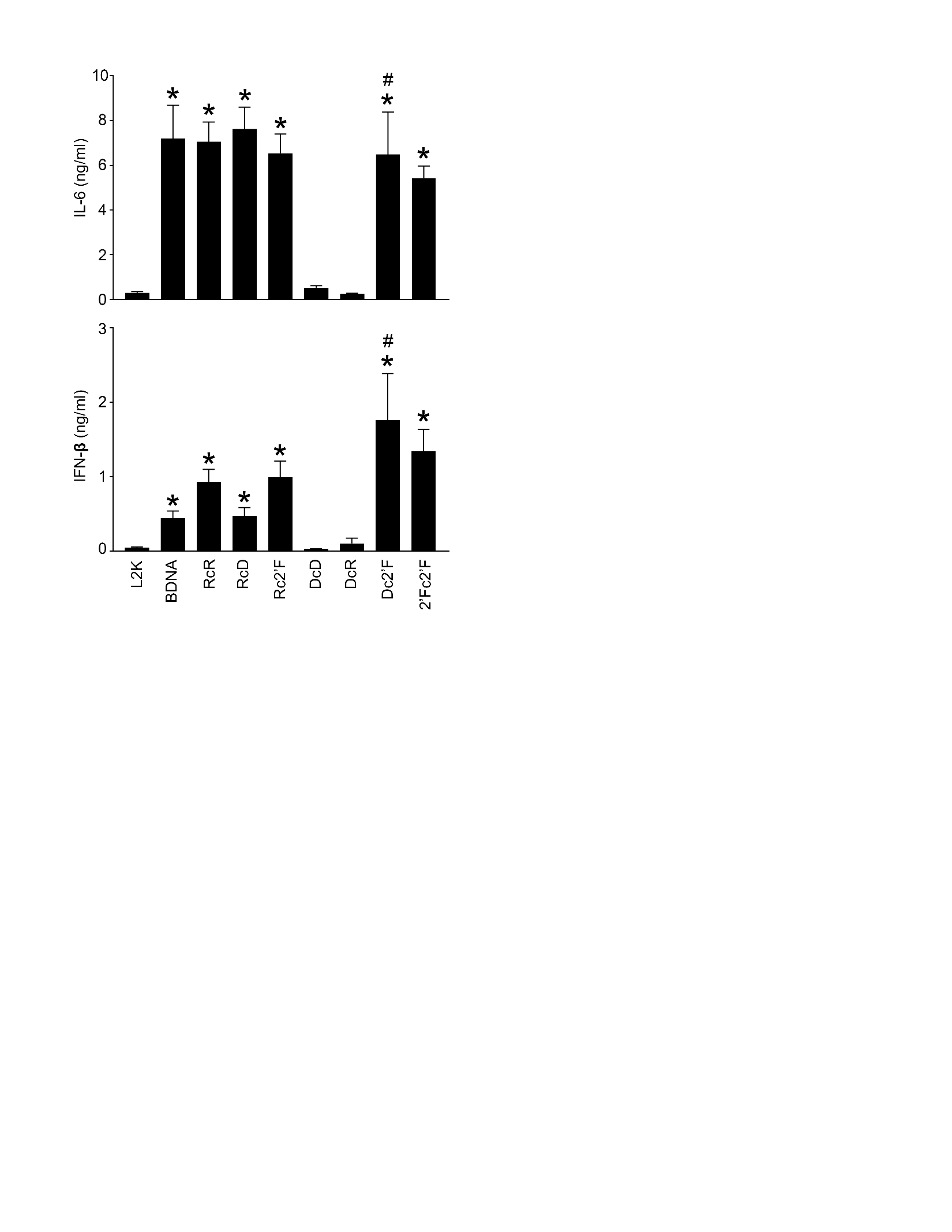
**

**Supporting Figure S4:** Fully modified NANPs (2’Fc2’F) stimulate IL-6 and IFN-β production. Microglia were transfected with 5 nM NANPs using L2K for 4 hours and cell supernatants were collected 24 hours post transfection. Then cell supernatants were analyzed for cytokine production using specific capture ELISAs for IL-6 and IFN-β. Data are represented as standard error of the mean (SEM) for a minimum three independent experimental replicates. Asterisks indicate statistical significance compared to L2K and hashtags indicate statistical significance compared to DcR (Student’s t-test, p-value < 0.05).

**
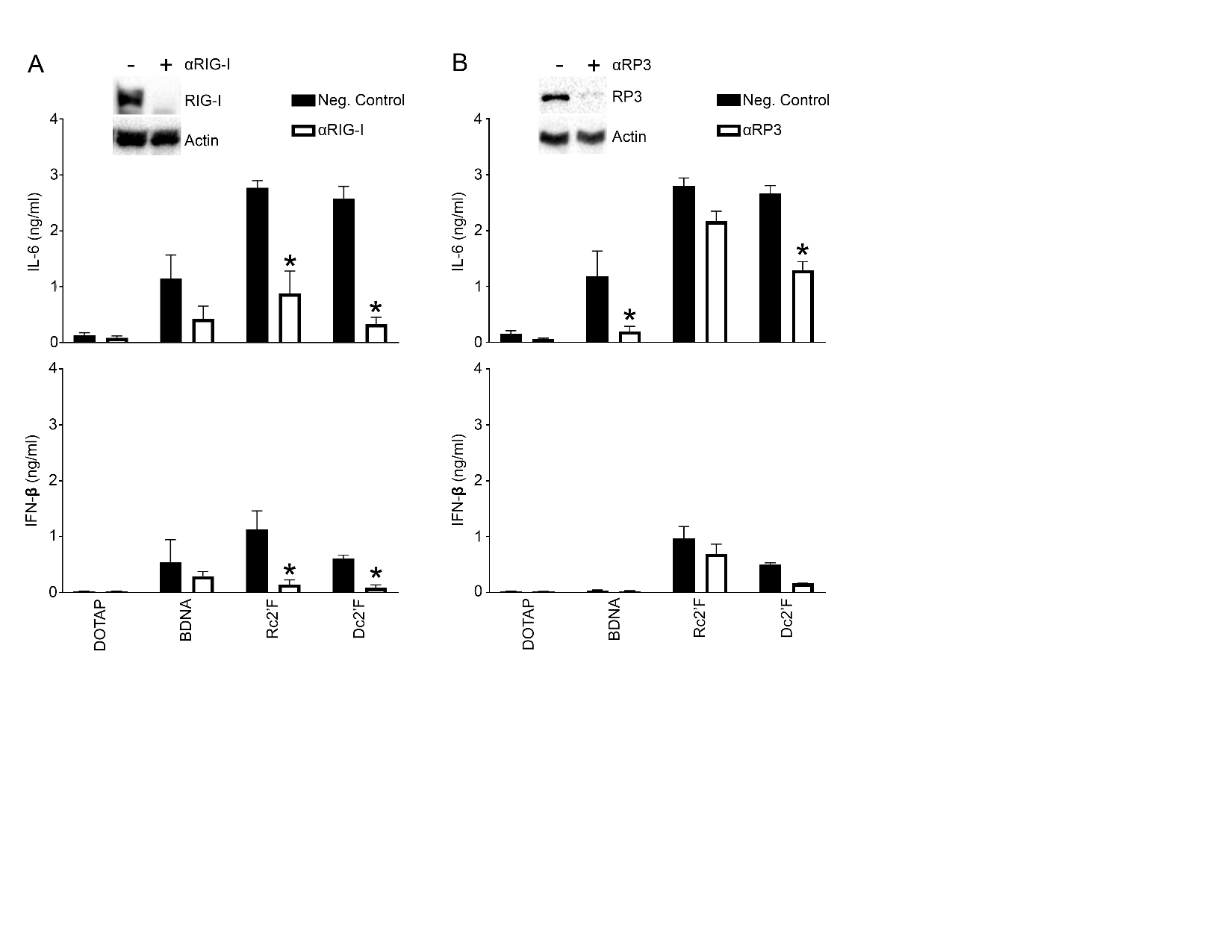
**

**Supporting Figure S5:** Microglia were treated with scrambled siRNA or siRNA targeting RIG-I (αRIG-I) at a final concentration of 5 nM for 24 hours (**A**). Microglia were treated with scrambled siRNA or siRNA targeting RNA polymerase III subunit A (αRP3) at a final concentration of 5 nM for 24 hours (**B**). Cells were placed in fresh media for 24 hours prior to transfection with 5 nM NANPs using DOTAP. Cell supernatants and lysates were collected 24 hours post transfection. Cell lysates were evaluated for either RIG-I (**A**) or RNA polymerase III subunit A (**B**) protein expression via immunoblot analysis. Cell supernatants were evaluated for IL-6 and IFN-β by specific capture ELISAs. Data are represented as the standard error of the mean (SEM) for two independent experimental replicates.
